# Spotted in the News: Using Media Reports to Examine Leopard Distribution, Depredation, and Management Practices outside Protected Areas in Southern India

**DOI:** 10.1371/journal.pone.0142647

**Published:** 2015-11-10

**Authors:** Vidya Athreya, Arjun Srivathsa, Mahi Puri, Krithi K. Karanth, N. Samba Kumar, K. Ullas Karanth

**Affiliations:** 1 Wildlife Conservation Society, India Program, Bengaluru, Karnataka, India; 2 Centre for Wildlife Studies, Bengaluru, Karnataka, India; 3 Wildlife Conservation Society, Global Conservation Program, Bronx, New York, United States of America; 4 Nicholas School of Environment, Duke University, Durham, North Carolina, United States of America; Centre for Cellular and Molecular Biology, INDIA

## Abstract

There is increasing evidence of large carnivore presence outside protected areas, globally. Although this spells conservation success through population recoveries, it makes carnivore persistence in human-use landscapes tenuous. The widespread distribution of leopards in certain regions of India typifies this problem. We obtained information on leopard-human interactions at a regional scale in Karnataka State, India, based on systematic surveys of local media reports. We applied an innovative occupancy modelling approach to map their distribution patterns and identify hotspots of livestock/human depredation. We also evaluated management responses like removals of ‘problem’ leopards through capture and translocations. Leopards occupied around 84,000 km^2^ or 47% of the State’s geographic area, outside designated national parks and wildlife sanctuaries. Their presence was facilitated by extent of vegetative cover- including irrigated croplands, rocky escarpments, and prey base in the form of feral and free-ranging dogs. Higher probabilities of livestock/human attacks by leopards were associated with similar ecological features as well as with capture/removals of leopards. Of the 56 cases of leopard removals reported, 91% did not involve human attacks, but followed livestock predation or only leopard sightings. The lack of knowledge on leopard ecology in human-use areas has resulted in unscientific interventions, which could aggravate the problem rather than mitigating it. Our results establish the presence of resident, breeding leopards in human-use areas. We therefore propose a shift in management focus, from current reactive practices like removal and translocation of leopards, to proactive measures that ensure safety of human lives and livelihoods.

## Introduction

Conserving large carnivores involves addressing unique socio-economic and cultural problems, seldom faced while dealing with other large mammals [1–3]. Protecting carnivore populations is therefore difficult, despite their charismatic appeal and flagship role in conserving natural ecosystems [4–6]. Wide-ranging carnivores are found across diverse habitats such as forests, agro-forests, grasslands, pasture lands, agricultural areas, and urban/semi-urban areas [7–10], their large geographic ranges often overlapping with human-dominated landscapes. The ability of predators like wolves (*Canis lupus*), coyotes (*Canis latrans*) and pumas (*Puma concolor*) to thrive in radically modified human-use habitats has been observed across the Americas [11–13]. Similarly, in parts of Africa, a suite of large carnivores share spaces with people [14–16].

Overlap of carnivore distributions and human-use areas increases human-predator interface, often leading to negative interactions [17]. Presence of carnivores in human-dominated landscapes may involve tangible economic costs from livestock depredation, and intangible opportunity costs of avoiding shared spaces/resources [7, 18, 19]. These negative interactions, loosely termed as ‘conflict’ could, in turn, have short-term consequences (e.g. retaliatory killing) as well as long-term impacts (e.g. reduced survival) on carnivores [20, 21]. Despite the threats and pressures they inflict upon each other, humans and carnivores do continue to share spaces, facilitated through a fractured yet complex and dynamic mechanism [22].

India offers an ideal opportunity for examining human-large predator interactions because it harbors around 23% of the world’s carnivore species, including large felids, canids and ursids, that share space with a dense human population of 1.2 billion people. Three large felid species, the Asiatic lion (*Panthera leo persica*), Indian tiger (*Panthera tigris tigris*) and Indian leopard (*Panthera pardus fusca*), occur in areas of high human population density (~400 people per km^2^; see Banerjee et al. [23] for lions; Athreya et al. [24] for leopards), a situation rarely seen elsewhere in the world. Although tigers are largely restricted to wildlife reserves, their numbers are currently increasing, and recent studies have documented tiger presence in human-use landscapes [25, 26].

Leopards are among the most adaptable and versatile large carnivores, occurring in a diversity of landscapes across India, wherever anti-hunting laws are enforced and cultural tolerance is higher [27–30]. However, very little is known about their ecology in human-dominated landscapes, with few studies [24, 30–32] generating such information. Although rural people who live in proximity to leopards and other carnivores do possess some empirical knowledge of their ecology [22], conservation and management of their populations based on rigorous scientific understanding remains grossly inadequate in India [33].

In this study, we focus on examining ecology of leopards at a large geographical scale, across Karnataka State in southern India. The State has a relatively high literacy rate, extensive cellphone networks, widespread television viewership, all of which contribute to rapid dissemination of information on human-leopard interactions in local and regional newspapers. Relying on data from media reports of leopard-related incidents, we first present a novel application of habitat occupancy modelling methods to map their distribution across the State. Simultaneously, we assess ecological, social, and biogeographic correlates associated with leopard presence in human-use landscapes. Second, based on media reports of leopard-related depredation incidents, we spatially map and estimate probabilities of attacks on livestock/humans by leopards; we also examine the social and management factors associated with such attacks. Finally, we critically evaluate present management interventions such as physical captures (removals) and translocations of ‘problem’ leopards. We discuss the consequences of these interventions for (i) persistence of leopards in human-use landscapes, (ii) reducing losses people suffer due to leopards, and (iii) implications for long-term conservation of leopard populations in these landscapes.

## Materials and Methods

### Ethics Statement

The study relied completely on information sourced from newspaper media reports, open source government websites and remotely acquired data. Animal care and use committee approval was not required.

### Study Area

**Fig 1:**
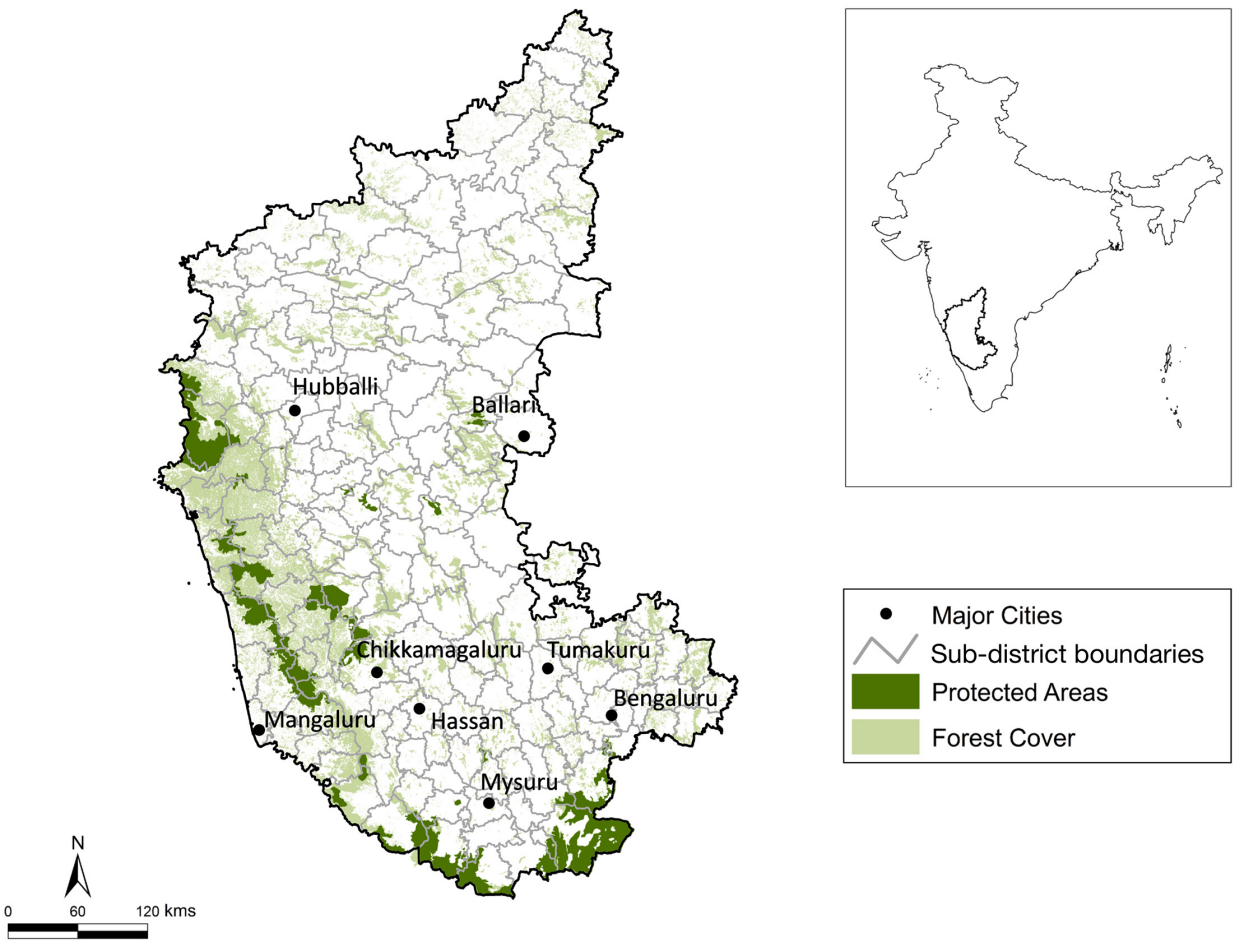
Study Area. Study area in Karnataka, southern India, with sub-district boundaries, protected reserves, forest cover, and eight major cities in the State. Inset: location of Karnataka within India.

The State of Karnataka constitutes nearly 6% (191,791 km^2^) of the country’s land area. Karnataka is divided into 30 districts (administrative units) and 175 taluks (sub-districts; Fig 1). The land cover is a heterogeneous mosaic of forests, agro-forest plantations, rocky escarpments, pasture lands, built-up urban areas, and large tracts of agricultural lands (see Das et al. [34] and Roy et al. [35]). The western and southern boundaries are hemmed with the tropical forests of the Western Ghats, which support source populations of large mammals like tigers, leopards, dholes (*Cuon alpinus*), sloth bears (*Melursus ursinus*), elephants (*Elephas maximus*), and a suite of large ungulate herbivores [36–39]. Following implementation of stringent wildlife protection laws in 1974, with a total ban on hunting of wildlife, earlier widespread hunting has declined substantially in the State [40].

In the years following 2010, there has been an increase in the number of leopard-related incidents being reported in the media. Most of these media reports are in the official State language (Kannada) or in English. The reports correspond to presence of leopards outside protected reserves, cases of alleged ‘straying’ of leopards into human-use areas, and attacks on livestock and, more rarely, on humans. The consistent trends in the locations and the nature of media reportage suggest the presence of leopard populations in human-dominated landscapes across Karnataka, as is the case in some parts of India with somewhat similar social contexts [24, 30]. The increasing interactions between humans and leopards have led to escalating management interventions such as physical removals and translocations of leopards by the State Forest Department, in response to pressure from the public.

### Data collection and processing

Survey assistants searched the internet, on a daily basis, for media reports related to nature, wildlife, and conservation issues from the study region. Data were collected for a 14-month period, from March 2013 to April 2014, from online editions of eleven publications- six English newspapers and five Kannada newspapers (see S1 File for the list of newspapers). We chose newspapers that cumulatively had the highest readership in the region, so as to optimize detections of leopard-related events. Since the news reports were searched and collected on a daily basis, online editions of each of these newspapers were scanned thoroughly to extract all articles of relevance to conservation issues in the region; reports related to leopards formed a subset of all articles collected in this manner. These data provided a snapshot sample of spatial distribution of leopards in the State. This method also allowed us to obtain information from human-use landscapes across a relatively large geographic area, where other conventional methods such as camera-trap surveys or sign surveys are not feasible.

Media reports were segregated into direct sightings of leopards, accidental leopard captures in snares/wells/buildings, leopard mortalities (both natural and human-induced), leopard attacks on livestock/humans, and leopard capture/removal by wildlife managers for either captivity or translocation to other sites. In cases where the location of the incident was reported, we assigned spatial coordinates based on the names of reported village, sub-district and district. Multiple reports of the same incident were combined and treated as unique records. For example, a unique record could potentially be a case that involved multiple news reports of a human attack by leopard, followed by the subsequent capture and translocation of the animal. We restricted the samples to only include reports of leopard-related incidents outside national parks and wildlife sanctuaries (henceforth, ‘protected reserves’) of the State.

### Spatial patterns of leopard distribution

We first sought to examine patterns of leopard distribution and factors associated with their occurrence across the State, outside protected reserves. Assessments were made at the level of each sub-district, since this was the finest resolution at which spatial locations could be unambiguously assigned. It is likely that we did not ‘detect’ the presence of leopards in certain areas due to ‘non-detection’ via media reports. In order to address this key issue of imperfect detection in the sampling process, we applied an occupancy sampling framework, which accounted for partial detectability [41, 42]. We treated the 175 sub-districts in the State as independent ‘sites’ (average size of sub-district = 1093 km^2^; range = 225–2796 km^2^). These areas are much larger than typical leopard home-ranges, thus ensuring we measured true occupancy [42]. Although data were collated for a 14-month period (March 2013 to April 2014), we chose a 12-month subset of the data (May 2013 to April 2014) for assessing distribution, so as to avoid including information from two months (March and April) twice. We used each of the 12 months as temporal replicates. Therefore, for each sub-district and in each month, detection of one/more leopard-related reports was assigned a value of ‘1’ and non-detection of leopards was assigned a value of ‘0’. For the purpose of this analysis, we make two key assumptions: (i) the overall spatial distribution of leopards in the State did not change significantly during the 12 months, and (ii) the true leopard occupancy status of any sub-district did not change- from occurrence to non-occurrence or vice versa- or changed randomly, during the study [43].

We estimated detection probability (*p*) and probability of leopard occurrence (Ψ) using the standard single-season occupancy model [41]. Here, Ψ is the probability with which a sub-district is occupied by leopards, and we interpret *p* as a combined probability of (i) a media publication detecting a leopard-related incident in a sub-district, and (ii) a leopard-related incident reported in media, being detected during our systematic internet searches. We followed a two-step process to estimate the key parameters. First, we fixed a general model for Ψ with a set of covariates that could potentially influence leopard occupancy at the sub-district scale. We modelled *p* against this model by varying combinations of covariates likely to influence detectability. We selected the most parsimonious model for detection probability based on Akaike’s Information Criterion (AIC; [44]). In the second step, we modeled occupancy probability (Ψ) by fixing covariates for detectability from the best-supported model in the previous step. We estimated occupancy (Ψ) by testing a set of candidate covariate models and chose the most suitable model based on AIC values and the relative model weights.

### Covariates for occupancy and detection probability

Probabilities of leopard occurrence and detection were likely to vary as function of ecological, biogeographic and social factors. Based on current knowledge of leopard ecology in human-dominated landscapes [24, 31, 32, 45, 46], we predicted that at the level of each sub-district, the density of livestock (cow, buffalo, goat, sheep, pig and stray cattle), density of dogs (owned and unowned), area under vegetation cover, area covered by rocky escarpments, and presence/extent of irrigated crop fields would be positively associated with leopard presence. In addition to these, we tested the influence of annual rainfall per sub-district (wet/dry areas could have different effects) and the size of the sub-district (to account for unequal sizes of sampling units) on leopard occurrence. Detailed descriptions of occupancy covariates, *a priori* predictions, and data sources are presented in Table 1.

**Table 1:** Descriptions of environmental and anthropogenic covariates used to model probabilities of leopard presence (Ψ) and probabilities of livestock/human attacks by leopards (Ψ_a_) outside protected reserves in Karnataka; *a priori* predictions of the direction of influence, and data sources.

Parameters	Covariates	Covariate description and *a priori* prediction	Source
Ψ	Vegetation Cover (vcov)	Land-cover types corresponding to 29 categories, which were likely to offer favorable vegetation cover, were re-classified (see S1 File for the list of land-cover types). The area under vegetation cover in each sub-district was computed. Predicted influence: Positive	Indian Institute of Remote Sensing, Govt. of India*
Ψ, Ψ_a_	Irrigated Cultivation (irrg)	Irrigated agricultural lands (with tall crop cover) are potential leopard habitats. Such areas have a higher value of mean Normalized Difference Vegetation Index (NDVI) during dry months, implying prolonged periods of standing crop-cover. Average NDVI values were calculated for cultivated areas in each sub-district. Predicted influence: Positive	MODIS/TERRA MOD13Q1 Vegetation Indices^+^; NDVI acquired for dry months (April-May 2013)
Ψ	Rocky Escarpments (rock)	Boulders, rocky hillocks and escarpments serve as ideal habitat refuges for leopards. Areas of boulders/rocky escarpments in each sub-district were computed. Predicted influence: Positive	Resourcesat-1 LISS III, Wasteland Map of India (2008–09), Govt. of India^$^
Ψ, Ψ_a_	Dog Density (dogs)	Dogs are a major component of leopard diet outside protected reserves. Sub-district-level densities of dogs (domestic, feral/semi-feral/free-ranging) were computed. Predicted influence: Positive	All India Livestock Census 2012, Govt. of India
Ψ, Ψ_a_	Livestock Density (lstk)	Leopard diet outside protected reserves is known to include livestock. Sub-district-level densities of livestock (cow, buffalo, goat, sheep, pig and stray cattle) were computed. Predicted influence: Positive	All India Livestock Census 2012, Govt. of India
Ψ	Rainfall (rain)	Dry/wet areas may favor leopard presence differently. Annual rainfall in each sub-district was used as an indicator. Predicted influence: Unknown	Annual Rainfall Report, 2013; Directorate of Economics and Statistics, Govt. of Karnataka
Ψ	Sub-district Area (area)	To account for variable size of sub-districts, the area of each sub-district was computed. Larger sub-districts may support relatively higher leopard populations. Predicted influence: Positive	Ministry of Communication and Information Technology, Govt. of India
Ψ_a_	Captures (capt)	Management interventions involving physical capture (removal) of leopards may be associated with attacks. Total number of captures from each sub-district was used. Predicted influence: Positive	Based on media reports
Ψ_a_	Releases (rels)	Management interventions involving translocation/release of leopards may be associated with attacks. Total number of releases into each sub-district was used.Predicted influence: Positive	Based on media reports

* Accessed from: http://bis.iirs.gov.in/

^+^ Accessed from: http://earthexplorer.usgs.gov/

^$^ Accessed from: http://bhuvan.nrsc.gov.in/gis/thematic/index.php

While modelling variation in detection probabilities, we used two covariates: (i) average distance from the sub-district centre to eight major cities (Ballari, Bengaluru, Chikkamagaluru, Hassan, Hubballi, Mangaluru, Mysuru, and Tumakuru; Fig 1)- the eight cities had local editions of newspapers that were most commonly detected during internet searches from Bengaluru. The predicted influence was negative, since news reports were gathered through internet sources from Bengaluru, and detectability (of media reports) was likely to be higher in sub-districts if they were closer to the major cities; (ii) size of sub-district was predicted to have a negative influence, implying that the chances of detecting leopard-related incidents would be lower in larger sub-districts. All covariates were screened for cross-correlations, and scaled and normalized prior to analyses. The list of candidate covariate models and model comparisons for the two-step process are in tables A1 and A2 in S1 Table.

### Identifying hotspots of livestock/human attacks by leopards

We assessed patterns of livestock/human attacks by leopards in the State under a probabilistic framework, using multi-state occupancy models [47, 48]. The choice of modelling approach was based on the premise that detectability of leopard presence, and livestock/human attacks by leopards, would be significantly influenced by the type of event (nature and degree of attacks), with the location of event, stakeholders involved, and level of sensationalization playing a major role. It was important, therefore, to delineate the difference in detectability for the two broad types of events reported (livestock/human attacks by leopards versus records of only leopard presence). The detection matrix for this analysis included ‘0’–non-detection of leopard-related reports, ‘1’–detection of reports documenting only leopard presence (state 1), and ‘2’–detection of reports documenting livestock/human attacks by leopards (state 2). We used the parameterization for multi-state occupancy described in MacKenzie et al. [48], where Ψ_p_ is the probability of leopard presence in a sub-district, and Ψ_a_ is the probability of occurrence of livestock/human attacks by leopards (subscripts ‘p’ and ‘a’ correspond to ‘presence only’ and ‘attacks’, respectively). The model allows for estimating three parameters for detectability:

*p*_*pp*_- probability of detecting reports of only leopard presence

*p*_*pa*_- probability of detecting reports of only leopard presence, although there may be livestock/human attacks in the sub-district

*p*_*aa*_- probability of detecting reports of livestock/human attacks by leopards

Detectability parameters were first modelled as a function of covariates, while retaining an intercept-only model for Ψ_p_ and Ψ_a_. Following this, Ψ_p_ and Ψ_a_ were modelled by fixing covariates for detectability from the best-supported model from the previous step. The list of candidate covariate models and model comparisons for the two-step process are in tables A3 and A4 of S1 Table. In modelling probability of livestock/human attacks by leopards (Ψ_a_), we used presence/extent of irrigated cultivation, dog density and livestock density in each sub-district as covariates. Since irrigated cultivation offers refuge in human-use areas, and dogs and livestock are both sources of prey for leopards outside protected reserves, we expected these factors to influence probability of livestock/human attacks. In addition to these, we also modelled the total number of physical captures (removals), and releases of leopards in each sub-district. This was done in order to examine the influence and directionality of attacks, i.e., to determine if attacks were likely to occur in capture locations or at release locations. The average distance to major cities and size of sub-district were used as covariates for modelling sub-district level detectability. Additional details on occupancy covariates, data sources, and predictions are in Table 1.

We used a likelihood-based modelling approach implemented in software program PRESENCE v. 5.7 [49], for estimating spatial distribution of leopards, and for predicting hotspots of attacks on livestock/humans by leopards.

### Threats and Management Interventions

Information from media reports allowed us to examine monthly trends in reportage of livestock/human attacks. In order to assess threats to leopards outside protected reserves, and management interventions involving leopards, we used data from the entire 14 month period. Reports sometimes yielded information on presence of cubs (indicating presence of resident, breeding leopards), and the numbers of leopard mortalities with associated causes. Together, these data facilitated a detailed analysis of leopard ecology in human-use areas of Karnataka, and an assessment of potential threats to leopard persistence.

We were particularly interested in examining management interventions that involved physical captures, followed by translocations and releases of ‘problem’ leopards. Therefore, we focused on obtaining information regarding reasons for physical capture, i.e., whether a leopard was captured after (i) only a sighting, (ii) livestock attack, or (iii) human attack. We also examined the post-capture fates of leopards, and ascertained the number of individuals that were translocated/released, held in captivity (zoos), or died in the translocation process. In cases where the locations of capture and corresponding release could be determined with certainty, translocation distances were calculated.

## Results

Our internet searches over 14 months yielded 408 media reports of leopards in human landscapes outside protected reserves. Collapsing multiple news articles related to unique events resulted in 245 records of leopard-related incidents. The subset of these data over a 12-month period (May 2013—April 2014) resulted in a total of 223 unique records of leopard-related incidents.

### Land-cover type, densities of non-wild prey influence leopard presence

Based on media reports, leopard presence was recorded from 68 of the 175 sub-districts of Karnataka (naive occupancy = 0.39). As per our prediction, the best-fit model for detectability (*p*) showed that the average distance to major cities [$\hat{\beta }$_city_($\hat{SE}$) = -0.48 (0.15)] and size of the sub-district [$\hat{\beta }$_area_ ($\hat{SE}$) = -0.24 (0.12)] negatively influenced detectability (A1 of S1 Table); we were more likely to detect media reports related to leopard occurrence closer to the major cities and from smaller sub-districts. We modelled occupancy probability (Ψ) after fixing the above covariates for detection probability. We tested 15 covariate models against the basic model [Ψ(.), *p*(city+area); A2 of S1 Table]. Since a single model did not receive full support from the data, we model-averaged across the top 11 models (with cumulative AIC weight >0.95) to obtain the final estimate of occupancy (Ψ).

**Fig 2:**
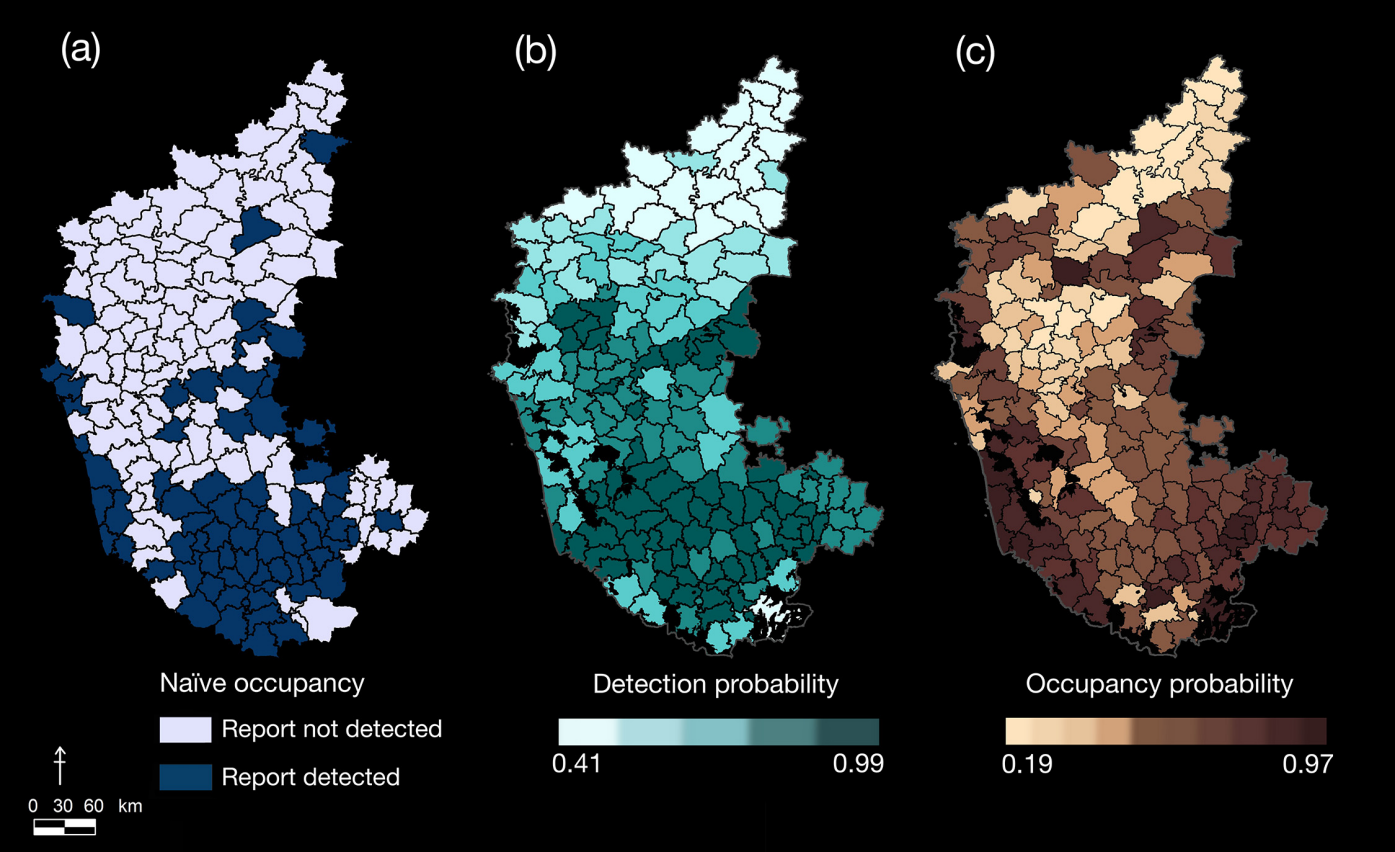
Distribution patterns of leopards in Karnataka. Spatial distribution of leopards outside protected reserves of Karnataka, based on analysis of media reports. The maps show sub-district-level estimates of (a) naive occupancy, (b) detection probabilities, and (c) probabilities of occupancy. Protected reserves have been clipped out from the predicted probability maps.

The density of dogs in each sub-district was positively associated with leopard presence. In terms of habitat/refuge, greater vegetation cover, rocky escarpments, and presence of irrigated crop fields favored leopard presence probabilities (Table 2). While density of livestock, annual rainfall, and size of sub-district appeared in the top 11 candidate models, their influence was unreliable (insignificant β-coefficients). The estimated average probability of leopard occupancy outside protected reserves in Karnataka was $\hat{\Psi }\;(\hat{SE}$) = 0.47 (0.01), with a relatively high detection probability [$\hat{p}\;(\hat{SE}$) = 0.83 (0.01)]. The highest probabilities of leopard presence were in sub-districts along the Western Ghats, the south-western, and south-central regions of the State (Fig 2).

**Table 2:** Estimates of β-coefficient values (respective standard errors in parentheses) for individual covariates associated with probabilities of leopard presence (Ψ) outside protected reserves in Karnataka, for the top 11 models from the candidate set (cumulative AIC weight >0.95). All models include fixed covariates for detectability [*p*(city+area)]. Subscripts for β-coefficients: int- intercept; vcov- vegetation cover; irrg- irrigated crop fields; rock- rocky escarpments; dogs- density of dogs; lstk- density of livestock; rain- annual rainfall; area- size of sub-district; city- average distance to eight major cities.

Models	${\hat{\beta }}_{\text{int}}\;(\hat{SE})$	${\hat{\beta }}_{\text{vcov}}\;(\hat{SE})$	${\hat{\beta }}_{\text{irrg}}\;(\hat{SE})$	${\hat{\beta }}_{\text{rock}}\;(\hat{SE})$	${\hat{\beta }}_{\text{dogs}}\;(\hat{SE})$	${\hat{\beta }}_{\text{lstk}}\;(\hat{SE})$	${\hat{\beta }}_{\text{rain}}\;(\hat{SE})$	${\hat{\beta }}_{\text{area}}\;(\hat{SE})$
Ψ(vcov+rock+dogs+lstk)	0.02(0.25)	0.43(0.32)	-	0.48(0.33)	0.87(0.41)	0.20(0.27)	-	-
Ψ(vcov+irrg+rock+dogs+lstk)	0.02(0.25)	0.74(0.43)	0.38(0.29)	0.53(0.33)	0.79(0.38)	0.14(0.28)	-	-
Ψ(vcov+dogs+lstk)	-0.06(0.22)	0.42(0.32)	-	-	0.85(0.40)	0.27(0.26)	-	-
Ψ(vcov+irrg+rock+dogs+lstk+area)	-0.08(0.26)	0.81(0.42)	0.41(0.29)	0.58(0.32)	0.69(0.36)	0.13(0.27)	-	-0.25(0.30)
Ψ(vcov+rock+dogs+lstk+area)	-0.04(0.26)	0.47(0.32)	-	0.51(0.32)	0.80(0.41)	0.20(0.26)	-	-0.17(0.30)
Ψ(vcov+irrg+dogs+lstk)	-0.06(0.23)	0.67(0.42)	0.30(0.27)	-	0.76(0.37)	0.25(0.27)	-	-
Ψ(vcov+irrg+rock+dogs+lstk+rain)	0.02(0.25)	0.74(0.44)	0.38(0.29)	0.52(0.33)	0.80(0.40)	0.13(0.31)	-0.03(0.31)	-
Ψ(vcov+dogs+area)	-0.07(0.23)	0.23(0.24)	-	-	0.89(0.44)	-	-	0.02(0.28)
Ψ(vcov+dogs+lstk+area)	-0.06(0.25)	0.42(0.32)	-	-	0.85(0.42)	0.27(0.26)	-	-0.004(0.28)
Ψ(vcov+irrg+rock+dogs+lstk+rain+area)	-0.09(0.25)	0.86(0.45)	0.42(0.28)	0.57(0.32)	0.71(0.38)	0.09(0.30)	-0.11(0.31)	-0.28(0.31)
Ψ(vcov+irrg+dogs+lstk+area)	-0.08(0.27)	0.68(0.42)	0.31(0.27)	-	0.73(0.39)	0.25(0.27)	-	-0.05(0.30)

### Management interventions and social factors associated with attacks on livestock and humans

We found reports of livestock depredation and human attacks by leopards from 51 of the 175 sub-districts. As predicted, the estimated probability of detecting reports of leopard presence-only [$\hat{p}$_*pp*_($\hat{SE}$) = 0.14 (0.01)] was significantly different from the probability of detecting reports of livestock/human attacks by leopards [$\hat{p}$_*aa*_($\hat{SE}$) = 0.82 (0.01)]. The top-ranked model for detectability suggested that the average distance to major cities negatively influenced both, the probability of detecting reports of leopard presence [*p*_*pp*_; $\hat{\beta }$_city_ ($\hat{SE}$) *=* -1.09 (0.47)], and the probability of detecting reports of livestock/human attacks by leopards [*p*_*aa*_; $\hat{\beta }$_city_ ($\hat{SE}$) *=* -0.26 (0.16); A3 of S1 Table]. The influence of average distance to major cities (β_city_) for *p*_*pa*_ had low reliability [$\hat{\beta }$_city_($\hat{SE}$) *=* -0.20 (0.19)].

We were unable to test covariate influences on probability of only leopard presence (Ψ_p_) since the occupancy models failed to converge. Therefore, we retained an intercept-only model for Ψ_p_, and modelled probability of livestock/human attacks (Ψ_a_) with covariate combinations. We tested 21 covariate models against the intercept-only model [Ψ_p_(.), Ψ_a_(.), *p*_*pp*_(city), *p*_*pa*_(city), *p*_*aa*_(city); A4 of S1 Table]. Again, a single model did not receive full support from the data. We therefore model-averaged across the top 8 models (with cumulative AIC weight >0.95) to obtain the final estimates of Ψ_a_. We found that probabilities of livestock/human attacks were most strongly associated with the number of leopard captures (removals) in each sub-district [top-model $\hat{\beta }$_capt_ ($\hat{SE}$) *=* 4.96 (2.50)]. Overall, the density of dogs and presence of irrigated crop fields were positively associated with livestock/humans attacks by leopards. The density of livestock and number of leopard releases (post-translocation) in each sub-district, however, did not have a significant influence. The model-specific β-coefficient values for individual covariates are provided in Table 3.

**Table 3:** Estimates of β-coefficient values (respective standard errors in parentheses) for individual covariates associated with probabilities of livestock/human attacks by leopards (Ψ_a_) outside protected reserves in Karnataka, for the top 8 models from the candidate set (cumulative AIC weight >0.95). All models include intercept-only model for presence [Ψ_p_(.)] and fixed covariates for detectability [*p*_*pp*_(city), *p*_*pa*_(city), *p*_*aa*_(city)]. Subscripts for β-coefficients: int- intercept; irrg- irrigated crop fields; dogs- density of dogs; lstk- density of livestock; capt- number of captures from each sub-district; rels- number of releases in each sub-district; city- average distance to eight major cities.

Models	$\hat{\beta }$_int_ ($\hat{\text{SE}}$)	$\hat{\beta }$_irrg_ ($\hat{\text{SE}}$)	$\hat{\beta }$_dogs_ ($\hat{\text{SE}}$)	$\hat{\beta }$_lstk_ ($\hat{\text{SE}}$)	$\hat{\beta }$_capt_ ($\hat{\text{SE}}$)	$\hat{\beta }$_rels_ ($\hat{\text{SE}}$)
Ψ_a_(irrg+dogs+capt)	1.66 (2.82)	0.38 (0.24)	0.91 (0.47)	-	4.96 (2.50)	-
Ψ_a_(dogs+capt)	1.80 (4.13)	-	0.94 (0.49)	-	4.52 (2.01)	-
Ψ_a_(irrg+dogs+capt+rels)	1.67 (2.84)	0.39 (0.25)	0.90 (0.47)	-	4.94 (2.45)	0.15 (0.55)
Ψ_a_(irrg+dogs+lstk+capt)	1.67 (2.82)	0.37 (0.26)	0.91 (0.47)	0.03 (0.25)	5.00 (2.62)	-
Ψ_a_(dogs+lstk+capt)	1.75 (3.47)	-	0.91 (0.49)	0.16 (0.22)	4.73 (2.47)	-
Ψ_a_(dogs+capt+rels)	1.82 (4.21)	-	0.94 (0.49)	-	4.51 (2.00)	0.03 (0.51)
Ψ_a_(irrg+dogs+lstk+capt+rels)	1.68 (2.83)	0.37 (0.26)	0.89 (0.47)	0.04 (0.25)	4.99 (2.61)	0.17 (0.56)
Ψ_a_(irrg+capt)	1.45 (2.49)	0.33 (0.23)	-	-	4.75 (2.13)	-

**Fig 3:**
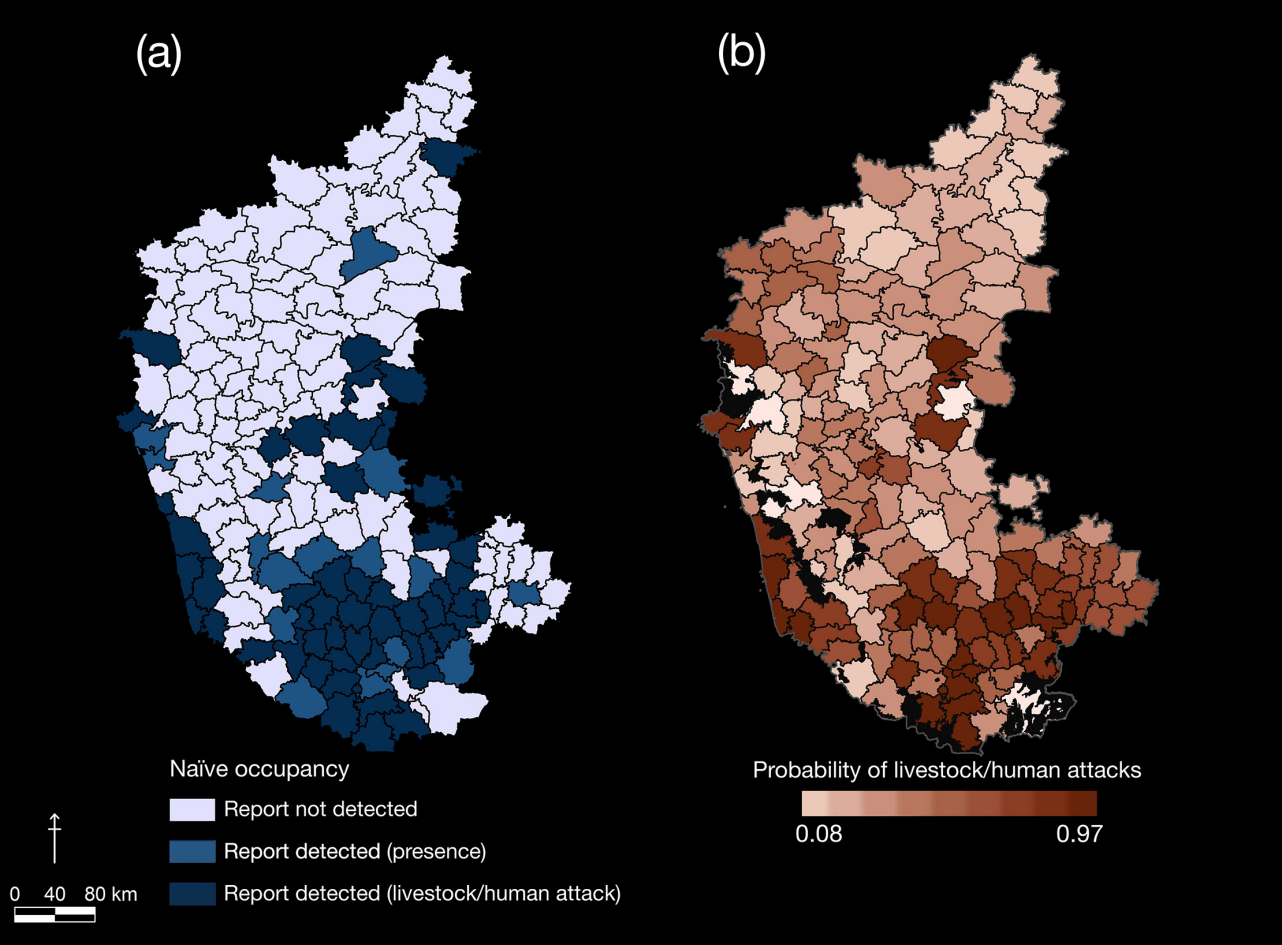
Patterns of livestock/human attacks by leopards in Karnataka. Hotspots of livestock/human attacks by leopards outside protected reserves of Karnataka, based on analysis of media reports. The maps show sub-district-level estimates of (a) naive occupancy and (b) probabilities of livestock/human attacks. Protected reserves have been clipped out from the predicted probability map.

We estimated the average probability of livestock/human attacks by leopards outside protected reserves in Karnataka at $\hat{\Psi }$_a_ ($\hat{SE}$) = 0.34 (0.02), while the sub-district level probabilities ranged from 0.08 (SE = 0.04) to 0.97 (SE = 0.001). The highest probabilities of livestock/human attacks by leopards were in sub-districts around the south-central region of the State (Fig 3).

### Conflict, threats and management interventions

During the 14-month survey, most reports of leopard-human interactions, frequently referred to as ‘conflicts’ in media reports, involved livestock depredation (83%). Of the 32 attacks on humans recorded during this period, three led to human deaths. The monthly trends in reports of livestock/human attacks did not show any distinct pattern, except for the higher number of attacks on humans in August 2013 (S1 Fig).

Overall, 19 cases of presence of leopard cubs (indicative of resident, breeding leopards) were reported from 15 sub-districts. In four of these cases, leopard cubs were trapped by the Forest Department in cages that were intended for capture of adult leopards that had attacked livestock. Following capture, these cubs were moved to captivity. We examined the cause of death in 34 cases of reported leopard mortalities. Of these, 26% was attributed to poaching, where leopard carcasses had evident signs like snares or gunshot wounds. There were equal number of reports (26%) where the cause of death could not be determined reliably. Road kills accounted for 24% of leopard deaths, and 9% were due to other accidents. There were some cases (6%) where leopards died following physical capture by the Forest Department either during translocation, or, from injuries sustained after being attacked by local residents. Deaths of leopards due to retaliatory killing by local people (6%) and natural deaths (3%) were also observed.

**Fig 4:**
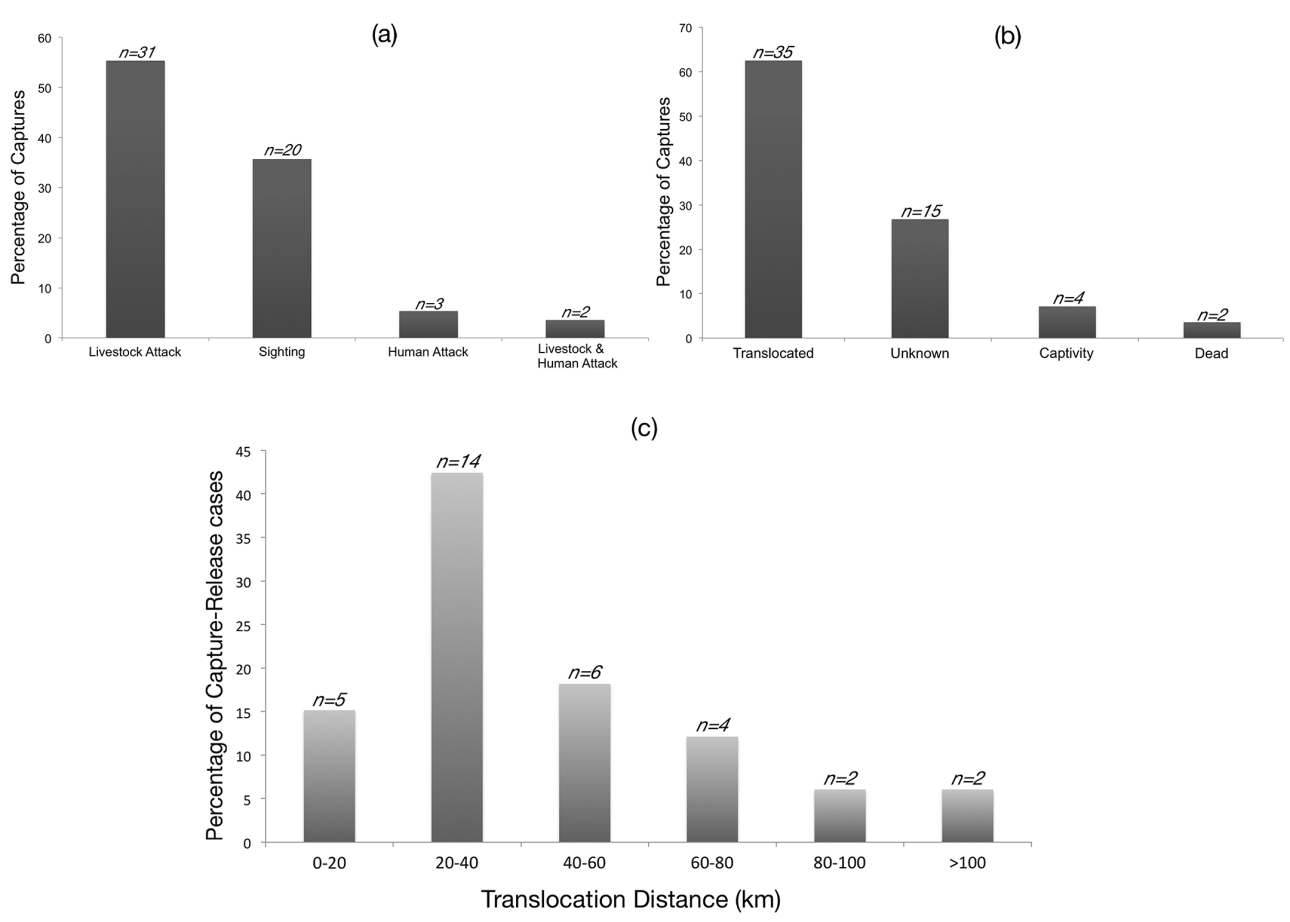
Reasons for leopard removal and post-capture fates of leopards. Percentage of leopard captures segregated based on (a) reasons for physical capture of individuals, and (b) post-capture fate of leopards trapped outside protected reserves of Karnataka. (c) Percentage of capture-release cases of leopards, segregated based on translocation distances (approximate distance between location of capture and location of release). The graphs correspond to data from media reports for 14 months (March 2013 to April 2014).

In order to understand the role of management interventions in dealing with ‘problem’ leopards, we specifically examined the cases involving physical captures, translocation, and releases. A total of 56 leopards were captured over 14 months, of which 91% of the captures were preceded by attacks on livestock (n = 31) or just sightings of leopards (n = 20) in human-use areas. Human injury or death was the reason for capture in five cases (Fig 4). Of the 56 leopards captured, >60% were translocated, and the fates of 27% of the individuals could not be ascertained from media reports (Fig 4). Translocation distances varied from as short as 5 km (where the leopard was released into the nearest forest patch) to ~190 km; although in most cases (42%), the release site was 20–40 km from the location of capture (Fig 4 and S2 Fig).

## Discussion

Conserving carnivores in India is a challenging task because eight species of large predators co-occur in landscapes with some of the highest densities of humans [23, 24, 50, 51]. The lack of ecological knowledge on human-wildlife interactions in human-use areas [33] preclude implementation of appropriate management decisions. The current ubiquitous presence of leopards in some human-dominated landscapes exemplifies this issue. Leopard occupancy within Karnataka was fairly high, with around 70 sub-districts (out of 175 sub-districts) showing >50% probability of presence (Fig 2). Furthermore, a minimum of 10% of sub-districts reported presence of cubs outside protected reserves, indicating a breeding population and not just transient individuals.

These results, although conservative, establish that there are resident leopard populations in human-dominated areas across Karnataka, repudiating widespread management notion that these are animals ‘straying’ from their ‘natural forested habitats’ that need to be captured and moved back. Leopards occupy approximately 47% of Karnataka’s area (~84,000 km^2^ excluding protected reserves). Even this may be an underestimate, because of incomplete media reportage. However, our estimates are in concordance with results of a nationwide survey by Karanth et al. [28], who estimated leopard occupancy at ~48% for Karnataka in 2007.

Presence of leopards in human-use areas presents two major conservation challenges. First, thereis need to mitigate cumulative risks to leopards from poaching, road accidents and retaliatory killings ([52, 53] this study). Second, there is an urgent need to minimize losses to local people by improving their animal husbandry practices, and by training and equipping wildlife managers to deal with human-leopard interactions more effectively.

### Factors facilitating leopard presence outside reserves

While the role of protected areas in conserving them is critical, several large carnivore species are not necessarily restricted to such protected wild areas [54]. For example, despite their preference for intact forest habitats, both jaguars (*Panthera onca*) and pumas can thrive outside protected areas, given adequate prey and cover conditions [55, 56]. Tigers in India have dispersed over great distances (>200 km), much of it through densely human-populated areas [25, 57]. Karnataka has ~40 000 km^2^ area under forest cover, of which <10 000 km^2^ area is under national parks or wildlife sanctuaries. Our study shows that additional cover and prey base available in unprotected forests, agro-forests, plantations and orchards (see S1 File), and rocky escarpments do play a crucial role in supporting a large leopard population outside these designated reserves.

India’s countryside, in some regions, supports high densities of feral, semi-feral, free-ranging, and domestic dogs [58]. In several human-use areas, dogs have been documented as prey species of leopards [59], and many other large predators [60]. Our findings suggest that dogs (as prey), rather than livestock, are more important in explaining leopard presence; free-ranging dogs perhaps assuming the role of primary non-wild prey. Our study corroborates findings of Athreya et al. [32], who found dogs to form around 40% of the biomass in leopard diet in a human-use landscape. In contrast, other felids like Asiatic lions [61] and tigers [62] prey substantially on livestock in human-use areas. Although smaller livestock species like goat and sheep do contribute to leopard diet [32], it is likely that they are better protected by their owners, as compared to domestic or free-ranging dogs. Due to the limited scope of this study, we were unable to investigate factors such as herding and husbandry practices, which could be mediating and mitigating livestock depredation by leopards at finer spatial scales.

### Negative interactions in human-use areas

Mapping spatial patterns of human-wildlife interactions (conflicts) has been a useful analytical tool for identifying areas of high risk, prioritizing management resources, and in conservation planning [63–65]. The application of probabilistic modelling to predict conflict patterns presented in our study (Fig 3), using an analytical framework that accounts for partial detectability, is relatively new (e.g., Goswami et al. [66]).

We found a positive association between captures of leopards, presence/extent of irrigated crop lands and probabilities of livestock/human attacks by leopards. The probabilities were highest in the south-central sub-districts (covering Mysuru-Tumakuru-Hassan-Bengaluru districts), and along the Western Ghats of Karnataka (Fig 3). The south-central areas have large tracts of irrigated sugarcane fields, complemented by cultivation of oilseeds and millets. Athreya et al. [24] documented resident leopard populations, at high densities, in sugarcane fields of western Maharashtra (India). These areas in Karnataka have historically been strongholds of leopards, with frequent attacks on livestock. Their presence was known locally, but did not qualify as ‘news material’ for media (pers. obs., K. U. Karanth and N. S. Kumar). Unlike the case with Asian elephants (see Goswami et al. [67]), agricultural areas with tall crops plausibly play a surrogate rather than a subsidiary role in supporting leopard populations outside parks in India.

Overall, leopard attacks on humans were low, and accounted for <20% of leopard ‘conflict’ records reported during our study. Furthermore, recent studies of human-wildlife interactions around five wildlife reserves of Karnataka showed that very few households (6% of 1972 respondents) reported livestock losses and human injury/death due to leopard attacks [68]. We believe that livestock losses could be mitigated with proactive measures that focus on assisting farmers in better husbandry practises, issuing prompt and just compensation for losses, and large-scale awareness on the distribution patterns of leopards in human-use landscapes.

### Leopard removals, translocations and local perceptions

We found that leopard captures were positively associated with probabilities of livestock/human attacks. Implicitly, this means removals are higher in sub-districts with more cases of depredation by leopards. But ~40% of leopard captures were made without any conflict, based just on leopard sightings. This substantiates a reverse mechanism, whereby removal of leopards from a sub-district is also increasing local conflict situations [69], either because other leopards are recolonising these areas, or, translocated leopards are homing back. Currently, the only methods of dealing with leopards in human-use landscapes in the State appears to be their translocation into protected reserves, and official payment of compensation losses due to leopard depredation. Karnataka had very few reported human attacks prior to 2010 (pers. obs., K. U. Karanth and N. S. Kumar; Chief Wildlife Warden, 2010, pers. comm. with V. Athreya). Aggressive removals might, in fact, be responsible for the subsequent sharp increase in attacks on livestock and more rarely, on humans. Such removals are largely a consequence of media and political pressure on managers, which builds up after occasional sightings of leopards. Unlike in the past, however, such information is then shared widely and rapidly using cellphone technology, leading to public anxiety and pressure. Further, removal of leopards merely after a sighting, catalyzed by media reportage and pressure, appears to be lowering people’s traditional socio-cultural tolerance of leopard presence, in the absence of any attacks. In the long run, such lowering of acceptance of wildlife in shared spaces would lead to a decline in potential leopard habitats, areas available for leopard population recovery, and a substantially smaller overall population size in a species that is still threatened and has suffered massive contraction of its global range [27].

Although leopard translocations have been successful in some parts of Africa [70, 71], this could be attributable to relatively low human populations (4 people/km^2^), high densities of wild ungulate prey, and other such local factors. In India, where human densities often exceed 400 people/km^2^, translocations of leopards have consistently failed [31, 69]. Within Karnataka, problem leopards that were removed from human-use areas were released into protected reserves; translocation distances ranging up to 190 km in some cases (see S2 Fig). Two leopards that were translocated during our study period, identified based on radio-collars placed on them, were found outside parks within 30–180 days of release (after our survey concluded).

Removal of ‘problem’ carnivores is difficult, expensive, involves high risks and its success in mitigating conflict has been mixed [72–74]. Since vacant territories are colonised by new individuals, sometimes from hundreds of kilometers away [75], large carnivore translocations often fail to eliminate the problem. Large felids, in particular, exhibit strong homing tendencies and often return to the site of their capture [30, 76, 77]. We believe such translocations have little scientific basis and are mostly unnecessary. They do not address the issue at the site of capture, and could well transfer the problem to the site of release. Furthermore, such captures often violate India’s wildlife laws which stipulate that leopards can be captured only when they have become dangerous to human life. In contrast, 91% of the leopard captures we recorded followed attacks on livestock or even mere sightings of leopards. Such practices are in fact, often in contravention of the guidelines on management of human-leopard conflict framed by the Indian government in 2011 [78].

### Conclusion

In this paper, we integrated information from media reports with an occupancy modelling framework to understand various aspects of leopard ecology and management in a relatively rapid and cost-effective manner. This approach could be applied to other studies for understanding past and present distributions of large carnivores in human-use landscapes, where there is strong presence of media and wide newspaper readership. We found that leopards are widely distributed outside protected reserves in Karnataka, that depredation is not as common as we expected, and management was not adequately equipped to deal with the presence of these predators. The debates on co-occurrence versus coexistence between humans and predators have sharply divided biologists, often on ideological grounds [6, 79–82]. Regardless of these debates, many recent studies provide basis to demonstrate that some large carnivores do share spaces with people in human-dominated landscapes. The case of leopards in Karnataka is one such example, and this knowledge needs to be integrated with current carnivore management strategies. Based on our findings, and a host of recent empirical studies, we propose a shift in management focus from reactive conflict redressal approach targeted at carnivore species, to more proactive measures that ensure safety of people and their property, enhance people’s acceptance of wildlife outside protected areas, and expand potential habitat for threatened species.

## Supporting Information

S1 Table A1. Model comparisons for estimating probability of detecting leopard presence (*p*) as a combined probability of (i) a media publication detecting a leopard-related incident in a sub-district, and (ii) a leopard-related incident reported in media, being detected during systematic internet searches. A2. Model comparisons to identify ecological and anthropogenic covariates associated with probability of leopard presence (Ψ), outside protected reserves in Karnataka. A3. Model comparisons for estimating probability of detecting leopard presence (*p*_*pp*_), probability of detecting leopard presence, although there may be livestock/human attacks (*p*_*pa*_), and probability of detecting livestock/human attacks by leopards (*p*_*aa*_). Detectability here refers to a combined probability of (i) a media publication detecting a leopard-related incident in a sub-district, and (ii) a leopard-related incident reported in media, being detected during systematic internet searches. A4. Model comparisons to identify covariates associated with probabilities of livestock/human attacks by leopards (Ψ_a_), outside protected reserves in Karnataka. (DOCX)

S1 Fig Monthly trends in reports of livestock/human attacks by leopards. Monthly trends in media reportage of livestock attacks, human attacks and other leopard-related incidents. The graph shows data based on media reports from 12 months (May 2013 to April 2014).
(TIF)

S2 Fig Leopard translocations in Karnataka. Schematic map of leopard translocations in Karnataka from March 2013 to April 2014, based on media reports. The lines represent links between 33 locations of leopard captures and corresponding sites of release. Inset: location of protected reserves in Karnataka.
(TIF)

S1 File A. List of newspapers and corresponding websites from where data were sourced for this study. B. List of land-cover types considered as potential leopard habitats in this study. (DOCX)
