# Supplementary material for: Spotted in the News: Using Media Reports to Examine Leopard Distribution, Depredation, and Management Practices outside Protected Areas in Southern India
Source: PLoS One. 2015 Nov 10;10(11):e0142647. doi: 10.1371/journal.pone.0142647 (PMC4640542; doi:10.1371/journal.pone.0142647)
Supplement: S1 Table — (DOCX) [file pone.0142647.s001.docx]

**S1 Table A1.** Model comparisons for estimating probability of detecting leopard presence (*p*) as a combined probability of (i) a media publication detecting a leopard-related incident in a sub-district, and (ii) a leopard-related incident reported in media, being detected during systematic internet searches.

| **Models** | **AIC** | **∆AIC** | **AIC weight** | **Model Likelihood** | **Parameters** | **Deviance** |
| --- | --- | --- | --- | --- | --- | --- |
| Ψ(vcov+irrg+dogs+lstk+area), *p*(city+area) | 1071.64 | 0 | 0.5959 | 1 | 9 | 1053.64 |
| Ψ(vcov+irrg+dogs+lstk+area), *p*(city) | 1072.68 | 1.04 | 0.3543 | 0.5945 | 8 | 1056.68 |
| Ψ(vcov+irrg+dogs+lstk+area), *p*(area) | 1077.24 | 5.6 | 0.0362 | 0.0608 | 8 | 1061.24 |
| Ψ(vcov+irrg+dogs+lstk+area), *p*(.) | 1079.22 | 7.58 | 0.0135 | 0.0226 | 7 | 1065.22 |
| Ψ(.), *p*(.) | 1088.49 | 16.85 | 0.0001 | 0.0002 | 2 | 1084.49 |

vcov- vegetation cover; irrg- irrigated crop fields; dogs- density of dogs; lstk- density of livestock; area- size of sub-district; city- average distance to eight major cities

**S1 Table A2.** Model comparisons to identify ecological and anthropogenic covariates associated with probability of leopard presence (Ψ), outside protected reserves in Karnataka.

| **Models** | **AIC** | **∆AIC** | **AIC weight** | **Model Likelihood** | **Parameters** | **Deviance** |
| --- | --- | --- | --- | --- | --- | --- |
| Ψ(vcov+rock+dogs+lstk), *p*(city+area) | 1067.82 | 0 | 0.1931 | 1 | 8 | 1051.82 |
| Ψ(vcov+irrg+rock+dogs+lstk), *p*(city+area) | 1067.93 | 0.11 | 0.1828 | 0.9465 | 9 | 1049.93 |
| Ψ(vcov+dogs+lstk), *p*(city+area) | 1068.98 | 1.16 | 0.1081 | 0.5599 | 7 | 1054.98 |
| Ψ(vcov+irrg+rock+dogs+lstk+area), *p*(city+area) | 1069.25 | 1.43 | 0.0945 | 0.4892 | 10 | 1049.25 |
| Ψ(vcov+rock+dogs+lstk+area), *p*(city+area) | 1069.5 | 1.68 | 0.0834 | 0.4317 | 9 | 1051.5 |
| Ψ(vcov+irrg+dogs+lstk), *p*(city+area) | 1069.67 | 1.85 | 0.0766 | 0.3965 | 8 | 1053.67 |
| Ψ(vcov+irrg+rock+dogs+lstk+rain), *p*(city+area) | 1069.92 | 2.1 | 0.0676 | 0.3499 | 10 | 1049.92 |
| Ψ(vcov+dogs+area), *p*(city+area) | 1070.17 | 2.35 | 0.0596 | 0.3088 | 7 | 1056.17 |
| Ψ(vcov+dogs+lstk+area), *p*(city+area) | 1070.98 | 3.16 | 0.0398 | 0.206 | 8 | 1054.98 |
| Ψ(vcov+irrg+rock+dogs+lstk+rain+area), *p*(city+area) | 1071.13 | 3.31 | 0.0369 | 0.1911 | 11 | 1049.13 |
| Ψ(vcov+irrg+dogs+lstk+area), *p*(city+area) | 1071.64 | 3.82 | 0.0286 | 0.1481 | 9 | 1053.64 |

vcov- vegetation cover; irrg- irrigated crop fields; rock- rocky escarpments; dogs- density of dogs; lstk- density of livestock; rain- annual rainfall; area- size of sub-district; city- average distance to eight major cities

**S1 Table A3.** Model comparisons for estimating probability of detecting leopard presence (*p_pp_*), probability of detecting leopard presence, although there may be livestock/human attacks (*p_pa_*), and probability of detecting livestock/human attacks by leopards (*p_aa_*). Detectability here refers to a combined probability of (i) a media publication detecting a leopard-related incident in a sub-district, and (ii) a leopard-related incident reported in media, being detected during systematic internet searches.

| **Models** | **AIC** | **∆AIC** | **AIC weight** | **Model Likelihood** | **Parameters** | **Deviance** |
| --- | --- | --- | --- | --- | --- | --- |
| Ψ_p_(.), Ψ_a_(.), *p_pp_*(city), *p_pa_*(city), *p_aa_(*city) | 1315.85 | 0 | 0.5559 | 1 | 8 | 1299.85 |
| Ψ_p_(.), Ψ_a_(.), *p_pp_*(city+area), *p_pa_*(city+area), *p_aa_*(city+area) | 1316.31 | 0.46 | 0.4416 | 0.7945 | 11 | 1294.31 |
| Ψ_p_(.), Ψ_a_(.), *p_pp_*(.), *p_pa_*(.), *p_aa_*(.) | 1327.98 | 12.13 | 0.0013 | 0.0023 | 5 | 1317.98 |
| Ψ_p_(.), Ψ_a_(.), *p_pp_*(area), *p_pa_* area), *p_aa_*(area) | 1328.12 | 12.27 | 0.0012 | 0.0022 | 8 | 1312.12 |

area- size of sub-district; city- average distance to eight major cities

**S1 Table A4.** Model comparisons to identify covariates associated with probabilities of livestock/human attacks by leopards (Ψ_a_), outside protected reserves in Karnataka .

| **Models** | **AIC** | **∆AIC** | **AIC weight** | **Model Likelihood** | **Parameters** | **Deviance** |
| --- | --- | --- | --- | --- | --- | --- |
| Ψ_p_(.), Ψ_a_(irrg+dogs+capt), *p_pp_*(city), *p_pa_*(city), *p_aa_(*city) | 1261.44 | 0 | 0.2878 | 1 | 11 | 1239.44 |
| Ψ_p_(.), Ψ_a_(dogs+capt), *p_pp_*(city), *p_pa_*(city), *p_aa_(*city) | 1262.01 | 0.57 | 0.2164 | 0.752 | 10 | 1242.01 |
| Ψ_p_(.), Ψ_a_(irrg+dogs+capt+rels), *p_pp_*(city), *p_pa_*(city), *p_aa_(*city) | 1263.37 | 1.93 | 0.1096 | 0.381 | 12 | 1239.37 |
| Ψ_p_(.), Ψ_a_(irrg+dogs+lstk+capt), *p_pp_*(city), *p_pa_*(city), *p_aa_(*city) | 1263.43 | 1.99 | 0.1064 | 0.3697 | 12 | 1239.43 |
| Ψ_p_(.), Ψ_a_(dogs+lstk+capt), *p_pp_*(city), *p_pa_*(city), *p_aa_(*city) | 1263.49 | 2.05 | 0.1033 | 0.3588 | 11 | 1241.49 |
| Ψ_p_(.), Ψ_a_(dogs+capt+rels), *p_pp_*(city), *p_pa_*(city), *p_aa_(*city) | 1264.00 | 2.56 | 0.08 | 0.278 | 11 | 1242.00 |
| Ψ_p_(.), Ψ_a_(irrg+dogs+lstk+capt+rels), *p_pp_*(city), *p_pa_*(city), *p_aa_(*city) | 1265.34 | 3.9 | 0.0409 | 0.1423 | 13 | 1239.34 |
| Ψ_p_(.), Ψ_a_(irrg+capt), *p_pp_*(city), *p_pa_*(city), *p_aa_(*city) | 1266.55 | 5.11 | 0.0224 | 0.0777 | 10 | 1246.55 |

irrg- irrigated crop fields; dogs- density of dogs; lstk- density of livestock; capt- number of leopard captures in each sub-district; rels- number of leopard releases in each sub-district; city- average distance to eight major cities
