# Supplementary figures and images for: Spotted in the News: Using Media Reports to Examine Leopard Distribution, Depredation, and Management Practices outside Protected Areas in Southern India
Source: PLoS One. 2015 Nov 10;10(11):e0142647. doi: 10.1371/journal.pone.0142647 (PMC4640542; doi:10.1371/journal.pone.0142647)

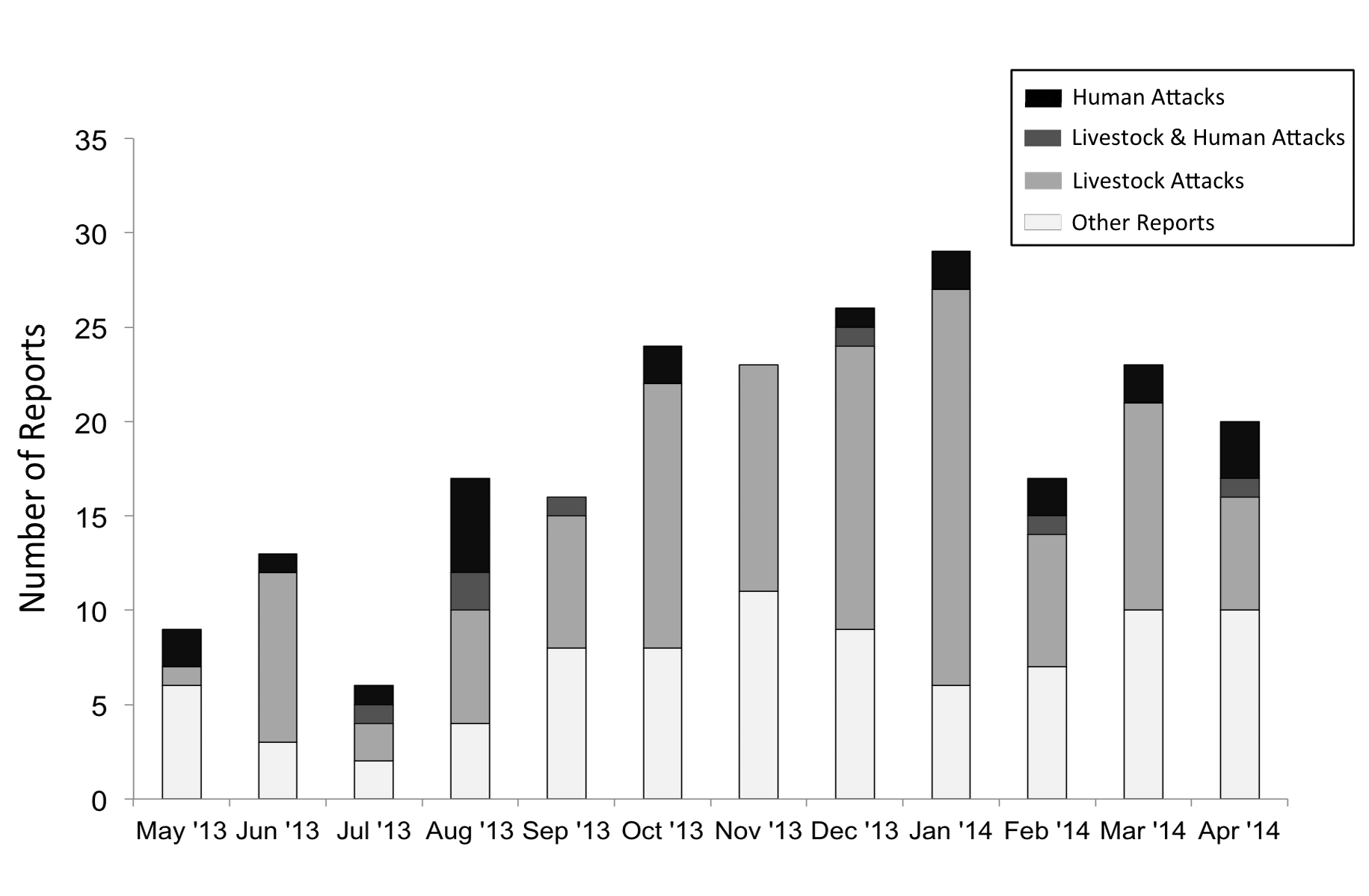

Supplement: S1 Fig — Monthly trends in media reportage of livestock attacks, human attacks and other leopard-related incidents. The graph shows data based on media reports from 12 months (May 2013 to April 2014). (TIF) [file pone.0142647.s002.tif]

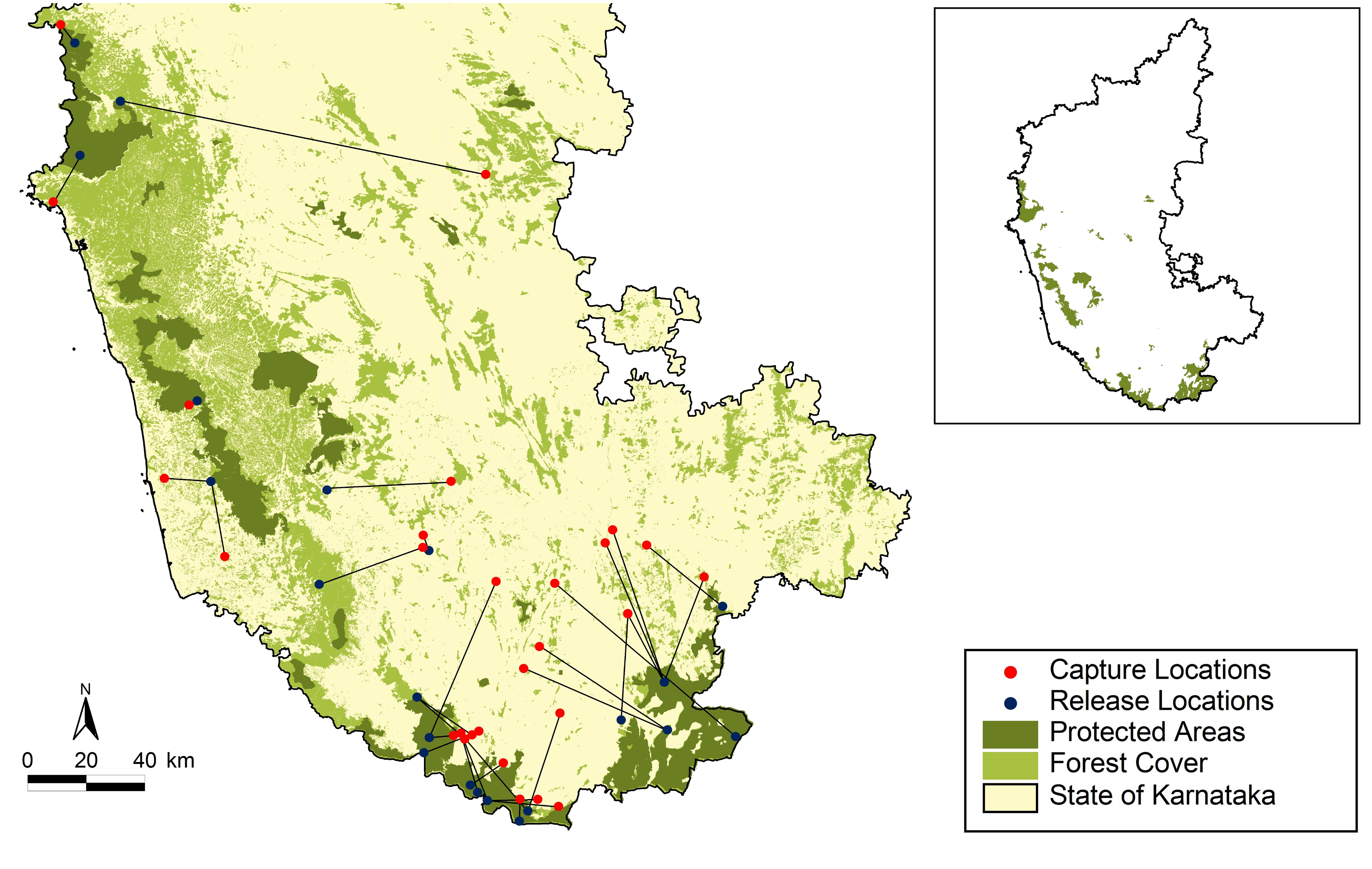

Supplement: S2 Fig — Schematic map of leopard translocations in Karnataka from March 2013 to April 2014, based on media reports. The lines represent links between 33 locations of leopard captures and corresponding sites of release. Inset: location of protected reserves in Karnataka. (TIF) [file pone.0142647.s003.tif]
