# Supplementary material for: Spotted in the News: Using Media Reports to Examine Leopard Distribution, Depredation, and Management Practices outside Protected Areas in Southern India
Source: PLoS One. 2015 Nov 10;10(11):e0142647. doi: 10.1371/journal.pone.0142647 (PMC4640542; doi:10.1371/journal.pone.0142647)
Supplement: S1 File — (DOCX) [file pone.0142647.s004.docx]

**A.** List of newspapers (and corresponding websites) from where data were sourced:

English newspapers:

1. The Hindu (http://www.thehindu.com)
2. Deccan Herald (http://www.deccanherald.com)
3. Times of India (http://timesofindia.indiatimes.com/home)
4. Deccan Chronicle (http://www.deccanchronicle.com)
5. The New Indian Express (http://www.newindianexpress.com)
6. Bangalore Mirror (http://www.bangaloremirror.com)

Kannada newspapers:

1. Kannada Prabha (http://www.kannadaprabha.com)
2. Vijayavani (http://www.vijayavani.net)
3. Prajavani (http://www.prajavani.net)
4. Udayavani (http://www.udayavani.com)
5. Vijaya Karnataka (http://www.vijaykarnatakaepaper.com)

**B.** Land-cover types corresponding to 29 categories considered as potential leopard habitats in this study: Evergreen Forest, Semi-evergreen Forest, Moist Deciduous Forest, Dry Deciduous Forest, Thorn Forest, Mixed Bamboo, Teak Plantations, Bamboo, Hardwickia Forests, Shola Forests, Kans, Forest Plantations, Eucalyptus Plantations, Acacia Plantations, Mixed Plantation, Degraded Forests, Woodland, Tree Savannah, Shrub Savannah, Scrub, Open Scrub, Dry Evergreen Scrub, Dry Deciduous Scrub, Euphorbia Scrub, Thorn Scrub, Shola Grassland, Orchards (Mango/Coffee plantations), Rubber Plantations, and Almond Plantations (see Roy et al. 2015 for detailed descriptions of the land-cover types).

**Reference**

Roy PS, Behera MB, Murthy MSR, Roy A, Singh S, Kushwaha SPS, et al. (2015) New vegetation type map of India prepared using satellite remote sensing: Comparison with global vegetation maps and utilities. Int J Appl Earth Obs 39: 142-159.
